# Insights into the roles of local translation from the axonal transcriptome

**DOI:** 10.1098/rsob.120079

**Published:** 2012-06

**Authors:** Alessia Deglincerti, Samie R. Jaffrey

**Affiliations:** 1 Graduate Program in Neuroscience, Weill Cornell Graduate School of Medical Sciences of Cornell University, 1300 York Avenue, New York, NY 10065, USA; 2 Department of Pharmacology, Weill Medical College of Cornell University, 1300 York Avenue, New York, NY 10065, USA

**Keywords:** axonal transcriptome, functions of local mRNA translation, microarray analysis

## Abstract

Much of our knowledge on the roles of intra-axonal translation derives from the characterization of a small number of individual mRNAs that were found to be localized in axons. However, two recent studies, using large-scale approaches to provide a more comprehensive characterization of the axonal transcriptome, have led to the discovery of thousands of axonal mRNAs. The apparent abundance of mRNAs in axons raises the possibility that local translation has many more functions than previously thought. Here, we review the recent studies that have profiled axonal mRNAs and discuss how the identification of axonal transcripts might point to unappreciated roles for local translation in axons.

## 2. Introduction

A major challenge in axonal biology has been to determine which aspects of axonal function depend on local translation and to identify the transcripts that mediate these effects. This goal originated 40 years ago, when several studies suggested that local protein synthesis may be a feature of developing axons. These studies used UV absorption and electron micrographic experiments on embryonic neurons to show that axons contain RNA and ribosomes [1–3]. Subsequent studies showed that specific mRNAs were present in axons and growth cones [4], and that regenerating axons from adult rat sensory neurons exhibited growth cone retraction after axonal application of the protein synthesis inhibitor cycloheximide [5]. However, the evidence linking intra-axonal protein synthesis to signalling pathways was established by studying the chemotropic responses to guidance cues [6].

Since then, more functions for local translation in axons have been uncovered, but progress in the field has been limited by the relatively small number of mRNAs identified in axons. Recent microarray analyses of the axonal transcriptome [7,8] have helped redefine the repertoire of mRNAs in axons by showing that the axonal transcriptome is much more complex and dynamic than previously thought. In turn, this suggests that our understanding of local translation and its functions needs to be revised to account for the characterization of novel classes of axonal mRNAs. This review will focus on the transcriptome-wide studies that have profiled axonal transcripts over the past 2 years and the new insights they offer on the roles of local translation in axons. For a detailed overview of the history of the field of intra-axonal protein synthesis and a broad discussion of various aspects of mRNA localization and translation in axons, the reader is referred to several excellent reviews on the topic that have been recently published [9,10].

## 3. Known functions of local translation

### 3.1. Guidance cue responses

Over the past decade, numerous studies have demonstrated that local translation of axonally localized transcripts is important for various functions of developing axons, including axon growth and pathfinding, as well as retrograde signalling (table 1). As mentioned earlier, local protein synthesis was implicated in mediating the responses of growth cones to several guidance cues, such as semaphorin 3A (Sema3A), netrin-1 and brain-derived neurotrophic factor (BDNF) [6]. In each of these cases, turning responses and other morphologic changes to growth cones were blocked by selective application of protein synthesis inhibitors to axons. Local translation was subsequently shown to be required for the adaptation responses of cultured *Xenopus* spinal neuron growth cones in gradients of extracellular guidance cues [39]. Growth cones undergo cycles of desensitization and resensitization to a specific guidance cue, and inhibition of local protein synthesis prevents resensitization from occurring [39].

**Table 1.** Identified roles for intra-axonal mRNA translation.

function of local translation	associated transcript(s)
chemotropic response to guidance cues	*RhoA* [11], *β-actin* [12–14], *cofilin* [15]
axon elongation	*PAR-3* [16], *β-thymosin* [17], *ATP5G1* [18], *COXIV* [19], *ALCAM* [20]
axon branching	*β-catenin* [21]
axon maintenance	*IMPA1* [22]*, Lamin *β** [23], *ATP5G1* [18], *COXIV* [24], *Cpg15* [25]
axon/neurite regeneration	*RPL4* [26]*, Importin *β** [27,28], *RanBP1* [28], *Vimentin* [29]*, NF-L, NF-M, NF-H* [30]
retrograde signalling: neuronal survival	*CREB* [31], *STAT3* [32]
retrograde signalling: tissue patterning	*Smad 1/5/8* [33]
synapse formation	*CEBP-1* [34], **α*-tubulin* [35], *Sensorin* [36]
unknown function	*Odorant receptors* [37]*, KOR1* [38]

Subsequent studies showed that local translation of specific axonally localized transcripts accounted for the protein synthesis requirements of these guidance cues. This was first demonstrated for Sema3A-induced growth cone collapse in axons of rat embryonic sensory neurons [11]. Transcripts encoding RhoA, a monomeric GTPase that regulates actin dynamics, were shown to localize to axons and shown to be translated in response to Sema3A signalling [11]. To definitively demonstrate that axonal RhoA transcripts, and not cell-body-localized transcripts, mediate this effect, a new technique termed ‘axon-specific knockdown’ was developed [40]. This approach, which uses application of siRNA exclusively to axons, showed that knockdown of *RhoA* mRNA selectively in axons impaired Sema3A-induced growth cone collapse, demonstrating that the axonal RhoA pool mediates the morphological responses of growth cones to Sema3A. Similarly, other studies have implicated local translation of β-actin, cofilin and Par3 in the responses to different guidance cues, such as netrin-1, Slit-2, nerve growth factor (NGF) and BDNF [12,13,15,16]. These studies demonstrated that local translation mediates the responses to numerous axon growth and guidance cues.

### 3.2. Retrograde signalling

More recent studies have indicated that local translation also has roles in other aspects of axonal signalling. For example, local translation has been implicated as a novel mechanism to convey signals from growth cones to the nucleus, thereby influencing gene transcription. This can be accomplished through local synthesis of transcription factors or adaptor proteins that are retrogradely trafficked to the cell body. Local synthesis of CREB, CEBP-1, STAT3, importins and SMAD transcription factors have all been linked to retrograde signalling mediated by NGF, BMP4 and nerve lesion [27,31–34]. These studies indicate that the consequences of local translation are not limited to localized responses but can extend to other subcellular compartments, such as the nucleus.

### 3.3. Control of axon-specific protein expression

Local translation may also be important for enabling axon-specific protein expression. For instance, local translation may facilitate the targeting of neuropeptides to axon terminals. Indeed, transcripts encoding vasopressin and oxytocin have been detected in nerve terminals of the posterior pituitary [41–43]. Local translation may also direct the expression of specific receptors in distal axons. For example, the κ-opioid receptor mRNA is localized to axons of mouse sensory neurons [38], and olfactory receptor mRNAs are found in distal axons of olfactory neurons [44,45]. Local translation may also regulate the timing of receptor expression in axons. For example, a reporter construct containing the 3′UTR of the guidance cue receptor EphA2 is upregulated in commissural axons only as the axons reach the midline [46]. Although it is not known whether endogenous *EphA2* transcripts are present or regulated in commissural axons, these findings raise the possibility that intermediate targets alter the chemotropic responses of axons by inducing local synthesis of guidance cue receptors. Together, these studies suggest a role for local translation in controlling the selective expression of specific proteins directly within growth cones.

### 3.4. Axonal regeneration

Although axonal localization and translation of mRNAs in developing neurons is well established, the same is not true for adult neurons. Some studies have identified mRNA in mature vertebrate neurons, including Mauthner cells [47], hypothalamic magnocellular neurons [42] and sensory neurons projecting to the olfactory bulb [48], but it is not clear whether axonally localized transcripts are a feature of most types of adult axons. Indeed, mRNA and rRNA levels disappear from axons of hippocampal neurons as they mature [49]. The mechanism by which mRNA and rRNA are lost is not currently understood. Although axons from adult neurons appear to contain reduced levels of mRNA and ribosomes than axons from embryonic neurons, *in vitro* studies from regenerating axons suggest that adult neurons possess the capacity to restore axonal localization of mRNA. Indeed, axons from adult rat sensory neurons preconditioned with nerve injury contain components of the translational machinery, including ribosomal proteins, rRNA and translation initiation factors both *in vivo* and *in vitro* [5]. In culture, these regenerating axons can synthesize new proteins even when severed from their cell bodies [5,26], and intra-axonal protein synthesis is required for proper axon regeneration [50]. Moreover, several studies have demonstrated the functional importance of intra-axonal translation of specific mRNAs in regenerating axons (table 1). Together, these data support the idea that adult axons use local mRNA translation after nerve injury to mediate axon regeneration.

## 4. Analysis of the axonal transcriptome reveals a complex population of localized mRNAs

Have these initial studies identified all the potential roles for local translation in axons? These studies have typically relied on the use of small-scale *in situ* hybridization [11] or small-scale axonal cDNA library screens [31] to identify axonal mRNAs. Because of the limited scale of these approaches, axonal transcripts may have been missed that would have otherwise pointed to novel roles for local translation in axonal biology. Thus, examination of axonal cDNA libraries has the potential to reveal novel functions of intra-axonal translation.

### 4.1. First-generation axonal mRNA libraries

Several studies (table 2) using both invertebrate and vertebrate neurons have established that a common set of transcripts is found in diverse types of axons. For example, cDNA libraries from squid giant axons [51,52] identified about 150 distinct transcripts by mRNA differential display. Sequence analysis of 27 of these mRNAs revealed a heterogeneous axonal mRNA population that encompassed transcripts for cytoskeletal components, translational machinery (including mRNAs for ribosomal proteins), nuclear-encoded mitochondrial proteins and signalling proteins [52]. Similar results were later obtained by the Twiss group who identified more than 200 different mRNAs in axons from injury-conditioned rat sensory neurons through hybridization to a cDNA microarray containing about 4000 rat cDNAs [53]. These transcripts encoded proteins involved in protein synthesis, intracellular transport, calcium regulation, metabolism and mitochondrial and cytoskeletal functions. cDNA libraries were also prepared from axons of cultured cortical cells which resulted in the identification of more than 300 transcripts [54]. Gene ontology analysis showed that transcripts with a role in translation, mitochondrial function, cytoskeleton regulation and intracellular transport were also enriched in these axons [54].

**Table 2.** A summary of profiling studies of axonal mRNA. The asterisk (*) denotes the number of transcripts identified based on the SAGE tags that match transcripts in currently available SAGE databases.

references	technique	cell type	no. transcripts identified
Gioio *et al.* [51,52]	mRNA differential display	squid giant axons	150 (axons)
Willis *et al.* [53]	microarray	injury-conditioned rat sensory neurons	200 (axons)
Taylor *et al.* [54]	microarray	E18 rat cortical neurons	308 (axons)
Andreassi *et al.* [22]	SAGE	neonatal sympathetic rat neurons	317(*) (axons)
Zivraj *et al.* [8]	antisense RNA+microarray	stage 24 (young) *Xenopus* retinal cells	286 (growth cones)
		stage 32 (old) *Xenopus* retinal cells	958 (growth cones), 5105 (axon shafts)
		E16 mouse retinal cells	2162 in growth cones
Gumy *et al.* [7]	random hexamer cDNA+microarray	E16 rat DRGs	2627 (axons)
		adult rat DRGs	2924 (axons)

More recently, serial analysis of gene expression (SAGE) has been used to characterize mRNAs in axons purified from neonatal rat sympathetic ganglia neurons cultured in Campenot devices [22]. SAGE results in the identification of short sequence tags that lie near the poly(A) tail of transcripts and that can uniquely identify individual mRNAs [55]. Almost 50 per cent of the tags from axonal RNA could not be mapped to known transcripts using current SAGE databases, making it unclear which transcripts they correspond to. However, the other 50 per cent identified a total of 317 axonal transcripts. Notably, half of these tags were derived from the 20 most abundant axonal mRNAs, suggesting that just a few mRNAs account for the majority of axonal mRNA. Consistent with earlier studies, these transcripts encoded mitochondrial proteins (30%), signalling proteins (20%), ribosomal proteins (15%), cytoskeletal proteins (5%) and other proteins involved in RNA metabolism and protein synthesis (5%). Interestingly, certain transcripts identified in axons from other types of neurons, such as CREB and cofilin, were not identified in axonal SAGE libraries, although they were also not present in the corresponding cell body SAGE libraries. One valuable feature of this study is that it allows the direct comparison of mRNA distribution in axons and cell bodies, thus making it possible to identify mRNAs that exhibit a high degree of enrichment in axons. These transcripts may be useful for indentifying sequence elements that target mRNAs to axons. In addition, an mRNA that is highly enriched in axons might have unique roles in axons compared with the one that is expressed at lower levels in axons.

Overall, these different libraries showed considerable overlap in the types of transcripts identified in axons, providing evidence that local translation may have common functions in axons from different types of neurons.

### 4.2. Second-generation axonal mRNA libraries

Two recent large-scale studies have used a microarray analysis to reveal a much more elaborate and complicated landscape of axonal mRNAs, identifying thousands of axonal mRNAs (table 2).

**Figure 1.**
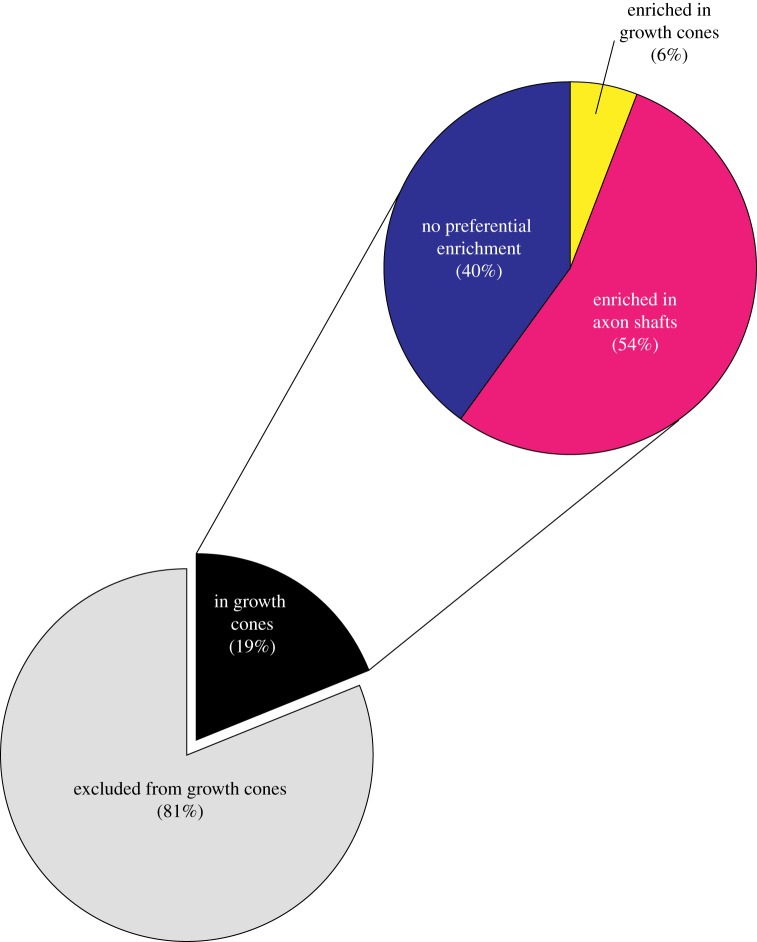
mRNA distribution between axons and growth cones (based on Zivraj *et al.* [8]). About one-fifth of the axonally localized mRNAs are found in growth cones, but only a small portion of these mRNAs (6%) are enriched in growth cones. These enriched mRNAs might have unique functions in growth cone biology (see text for details).

The first study not only identified axonal transcripts, but also identified transcripts in specific domains within axons [8]. This group used laser-capture microdissection coupled with microarrays to profile the transcriptome of growth cones and axon shafts from *Xenopus* retinal ganglion neurons [8]. Unlike previous axonal transcriptome studies, this approach distinguished between mRNAs that are enriched within neuronal growth cones from mRNAs that are enriched in axon shafts. Transcripts that are localized in axon shafts are likely to be translated and affect local processes within axon shafts. Thus, the division of the axonal transcriptome into growth cone and axon shaft-enriched pools may be valuable to predict potential roles of specific axonal mRNAs. The study identified 5105 transcripts that localize to axon shafts of stage 32 neurons and 958 transcripts that localize to the growth cones of the same type of neurons [8]. Of the mRNAs that are present in growth cones, only a minimal portion (6%, 58 transcripts) are enriched in growth cones, while the majority of the transcripts found in growth cones (54%, 523 transcripts) are enriched in axon shafts (figure 1).

What does this mean in terms of axonal biology and local translation? Differential analysis of the growth cone and axonal transcriptomes revealed that growth cones are enriched for transcripts encoding cytoskeletal and motor proteins, proteins involved in translation, transmembrane and cell surface receptors, nuclear proteins, secreted proteins, signalling proteins and metabolic proteins [8]. On the other hand, transcripts encoding proteins with roles in protein degradation, apoptosis, membrane trafficking, cell cycle and transcription factors are enriched in axon shafts. This apparent difference in mRNA localization and distribution within the periphery might underlie fundamental differences between the roles of local translation in axons and growth cones.

The idea that the differences in the axonal and growth cone transcriptomes have functional roles presupposes that translation can occur in both sites. Although translation is often thought to occur in growth cones, translation in axons has also been demonstrated [11]. However, it will be important to confirm that the newly identified axonal mRNAs are indeed translated in axon shafts. If these axonal mRNAs are not translated within axon shafts, then an alternative model could be that axons are storage sites for translationally silent mRNAs and that mRNAs need to be transported to growth cones to be activated. Intriguingly, a similar phenomenon has been observed in dendrites where upon stimulation, dendritic mRNAs translocate to activated synapses within spines, where local translation occurs [56,57].

The study by Zivraj *et al.* [8] also revealed that the composition of the growth cone transcriptome changes during neuronal maturation. Early embryonic *Xenopus* retinal ganglion growth cones (stage 24) contained 286 unique transcripts, compared with the 958 contained in growth cones of late embryonic (stage 32) [8]. Functional classification of the mRNAs from the two datasets showed that growth cone transcripts from many different functional categories were upregulated in older axons. The developmentally regulated, axonally localized transcripts may allow local translation to serve different roles at different stages of neuronal development.

This study further showed that an evolutionarily conserved core set of mRNAs is found in axons from neurons of different species. Of the 444 known growth cone transcripts identified in stage 32 *Xenopus* retinal ganglion neurons, 238 transcripts encoding homologous proteins were also found in growth cones from mouse retinal ganglion neurons of similar developmental stage. This suggests a moderate degree of conservation between the two systems that might point to the existence of conserved functions of local translation.

In the second study, distal axons from embryonic and adult rat sensory neurons were compared [7]. A microarray analysis revealed that 2627 transcripts localize to embryonic axons and 2924 to regenerating adult axons [7]. About 50 per cent of transcripts (1445 mRNAs) were present in both embryonic and adult axons, further supporting the idea that a core set of mRNAs localizes to axons, regardless of the developmental stage. An Ingenuity pathway analysis of the transcript population common to both types of axons revealed that both embryonic and adult sensory axons contained transcripts encoding mitochondrial proteins, proteins linked to neurological diseases and proteins involved in mRNA translation and protein synthesis. Surprisingly, cytoskeletal- and transport-related mRNAs as well as cell cycle mRNAs were a major component of the embryonic axonal transcriptome, whereas inflammatory and immune response mRNAs were present only in adult axons. These findings suggest that additional sets of mRNAs may be present at distinct stages of development to perform stage-specific axonal functions.

Although these studies identified a large population of axonal mRNAs, technical limitations may have prevented a full appreciation of the axonal transcriptome. In particular, the brain is highly enriched in transcripts that lack the poly(A) tail [58,59]. Signal-dependent polyadenylation of transcripts is thought to be a mechanism that regulates local translation in axons [60], which may indicate that transcripts lacking the poly(A) tail are likely to have important roles in mediating translation-dependent responses to signalling molecules in axons. Because the poly(A) tail is required for detection of transcripts in certain library approaches, such as SAGE [22] or cDNA synthesis using oligo(dT) primers as in Zivraj *et al.* [8], these approaches may miss important subsets of axonal RNAs. This might also explain differences in the apparent content of axonal RNA libraries that have been generated thus far, because different axonal populations may have different pools of polyadenylated mRNAs.

These studies demonstrated that the axonal transcriptome is complex and highly regulated. With the number of axonal transcripts jumping from hundreds to thousands of mRNAs, these studies allow broader conclusions about the major roles for local translation in axons. Some of these are discussed subsequently.

## 5. Not all axonal mRNAs are equal

A fluorescent *in situ* hybridization (FISH) analysis of transcripts localized to growth cones revealed that the signals for different transcripts have different sizes and density [8]. Axonal mRNA was shown to be localized in growth cones as discrete puncta, consistent with previous studies that showed the organization of mRNAs in granules [4,11,61]. For some mRNAs such as ephrin A3 and alpha tubulin, the puncta appeared as relatively small, dense puncta, while for other transcripts, such as RPS13, ferritin or ubiquitin C, the puncta were larger and more sparsely distributed.

The differences in the localization and size of individual axonal mRNAs suggest that they may be associated with distinct types of RNA granules. RNA granules contain RNAs, ribosomal subunits and various proteins such as translation factors, decay enzymes and RNA-binding proteins that may mediate different aspects of RNA regulation in neurons [62]. For instance, different types of RNA granules may mediate mRNA trafficking into growth cones, may serve as a reservoir to store mRNAs in translationally silent complexes or may mediate RNA degradation [62]. Alternatively, the different labelling patterns observed in FISH experiments may reflect differences between translationally active and inactive mRNAs. Different types of RNA granules have been defined by the presence of specific markers, such as Staufen, FMRP, zipcode-binding protein-1 (ZBP-1) and SMN for transport granules, TIA-1 and TIAR for stress granules and Dcp1a, RCK and GW182 for P-bodies [62]. Colabelling of the various mRNA puncta with granule-specific markers will be important to decipher the significance of differential mRNA localization and distribution in growth cones. Moreover, it will be interesting to analyse the granules that contain transcripts that are found only in axons and not growth cones. Identifying the properties of these granules might help to clarify how these mRNAs are excluded from growth cones.

The FISH study also raises questions about the localization of mRNA within the growth cone. All the mRNAs analysed primarily localized to the central domain of the growth cone, with some puncta occasionally found within the proximal portion of filopodia [8]. However, this would be in contrast with a recent report that found that both translational machinery components and newly synthesized protein are found at the tips of filopodia [63]. The observed absence of puncta along the length of filopodia may indicate the general exclusion of mRNA from filopodia, or it may reflect the resolution limits of the FISH procedure. FISH signals may derive from mRNAs within granules, where multiple copies of the transcript are packed together. FISH may not detect single copies of mRNA that may be released from storage granules upon activation of translation. Indeed, ultrastructural studies of neurons in culture suggest that RNA granules release mRNAs upon neuronal stimulation, potentially enabling translation of the released transcripts in filopodia [64].

## 6. Are nuclear-encoded mitochondrial proteins synthesized in axons?

One of the most abundant classes of axonal mRNAs is transcripts encoding mitochondrial proteins, suggesting that a major role of local translation is to provide proteins required for mitochondrial function. Although mitochondria are well known to generate ATP for various metabolic processes, they also provide metabolic intermediates used for amino acid synthesis [65], which may be important to support local translation. mRNAs encoding mitochondrial proteins account for almost 30 per cent of the axonal transcripts identified by SAGE [22], which represents a 1.5-fold relative enrichment in axons compared to cell bodies. Mitochondria contain 600–1000 unique proteins [66], of which only 13 are encoded by the mitochondrial genome [67]. The remainder are encoded in the nuclear genome and subsequently transcribed and translated in the cytosol prior to import into the mitochondria [68]. If the proteins in the mitochondria require replacement, then local translation could be particularly important for efficiently providing these proteins, and therefore preserving mitochondrial function. Indeed, Kaplan and co-workers [69] have shown that selective inhibition of axonal protein synthesis reduces axonal mitochondrial membrane potential.

Transcripts that encode mitochondrial proteins were among the first axonal transcripts that were identified in first-generation axonal mRNA libraries [51]. Subsequent work has established that several classes of mitochondrial proteins are synthesized in axons, including H^+^-ATP synthase and cytochrome *c* oxidase subunits, Hsp70 and Hsp90 (molecular chaperones that mediate protein import in the mitochondria), DNA polymerase γ and mitochondrial ribosomal proteins [69].

Although not all mitochondrial proteins seem to have transcripts that localize to axons [8], this could reflect a preferential axonal enrichment for proteins that exhibit the highest degree of turnover in mitochondria.

## 7. Can axons make or repair ribosomes?

Another class of highly abundant axonal RNAs is transcripts encoding ribosomal proteins. Although transcripts encoding ribosomal proteins are prevalent in cells, an analysis of the SAGE data indicates that these transcripts are 2.5-fold enriched in axons and comprise 15 per cent of all axonal transcripts. The mature ribosome consists of a small (40S) and a large (60S) subunits that comprise over 80 proteins and four rRNAs (5S, 5.8S, 28S and 18S) [70]. Transcripts for most ribosomal proteins can be found in axons. Thus, a significant portion of local translation in axons is likely to be dedicated to maintaining an adequate supply of ribosomal proteins.

The idea that ribosomal proteins may be synthesized in axons is contrary to current views of ribosome assembly, in which ribosomal proteins are imported into the nucleus for assembly into mature ribosomal subunits [71]. The ribosomes that are localized to axons are assumed to have been transported there as fully assembled subunits. Indeed, ribosomes have been observed to be trafficked into axons as a major constituent of RNA granules [72].

The discovery of extensive ribosomal protein transcripts in axons raises the possibility that axons possess the capacity to use locally synthesized ribosomal proteins. These proteins could be used for de novo ribosome assembly in axons or for more selective replacement of damaged proteins from otherwise intact ribosomes. However, ribosome assembly involves nearly 200 essential assembly factors [71] including ATPases, GTPases, kinases and other post-translational modifiers, which would need to be present in axons. Interestingly, the mRNAs for several nucleolar proteins involved in the synthesis and maturation of the ribosome, such as nucleolin, EMG1, GNL3 and Nola1–5, are present in axonal libraries, raising the possibility that some aspects of ribosome assembly or ribosomal protein replacement could occur in axons. It would be advantageous to extend the lifetime of a ribosome in axons, thus reducing the need for transport of new ribosomes from the cell body to the nerve terminals. Indeed, ribosomal subunits are thought to be unstable owing to their targeting by the ubiquitination pathway [73], and therefore may require replacement. Because of the intricate nature of the multi-subunit structure of the ribosome, it is unlikely that a ribosomal protein would spontaneously associate with the ribosome. However, the ability of axons to support ribosome assembly or repair remains to be established.

Interestingly, not all transcripts for ribosomal proteins have been identified in axons, raising the intriguing possibility that axonal ribosomes are slightly different from the classic cell body ribosomes. This could lead to ‘ribosomal specialization’, a phenomenon in which ribosomes of specific composition preferentially translate specific subsets of mRNAs [74]. Further analyses of axonal ribosomes through biochemical studies are needed to determine whether axonal ribosomes have a different protein composition compared to their cell body counterparts.

## 8. Why are transcripts for nuclear proteins localized to distal axons?

Almost 15 per cent of the axonal transcriptome comprises mRNAs encoding proteins that normally function in the nucleus, such as transcription and splicing factors, and transcriptional regulators [8]. It is possible that some of these transcripts encode proteins that have novel non-nuclear functions. In this case, they may act locally within axons to influence axonal properties. For example, Holt and co-workers [23] recently described a role for the intermediate filament protein lamin B2, normally found associated with the nuclear membrane, in promoting axon maintenance through local translation of its mRNA in *Xenopus* retinal ganglion neurons.

An alternative model to explain the prevalence of axonal mRNAs for nuclear proteins is that these transcripts are translated within axons, and are then retrogradely trafficked to the cell body to influence nuclear functions. Indeed, axons contain importins, adapter proteins that bind nuclear localization sequences (NLSs) in target proteins [75]. Importins couple bound proteins to dynein motors, thus enabling the retrograde trafficking of importin-bound proteins to the nucleus [27]. Therefore, many proteins that normally function in the nucleus could be retrogradely trafficked and translocated into the nucleus because they typically contain NLSs. Consistent with this idea, Cox *et al.* [31] reported that the transcription factor CREB is locally synthesized in embryonic dorsal root ganglion (DRG) axons and growth cones and retrogradely transported to cell bodies where it promotes neuronal survival. Subsequent studies have supported the idea that transcription factors can be locally synthesized and retrogradely trafficked to influence transcription. These include studies showing that CEBP-1 and STAT3 are locally translated in sensory axons after injury [32,34]. Similarly, SMAD1/5/8 is retrogradely trafficked from trigeminal neuron axons to nuclei to induce neuronal subtype specification [33]. The recent second-generation axonal libraries have identified several other axonally localized mRNAs for transcription factors, including TFIID, PAX2, TAF10, Myc2, STAT1, CREB3, SMAD1 and ATF [7,8], raising the possibility that local translation and retrograde trafficking of transcription factors may be a recurrent feature in axonal signalling.

In addition to transcription factors, other locally synthesized proteins may influence transcription and other nuclear functions. Indeed, mRNAs for DNA polymerase δ and its cofactor PCNA, histone H2 and H3 and other chromatin regulators (such as HMG-box proteins HMG17 and HMG2L1), splicing factors (in particular Sfrs2, Sfrs3, Syf2, Khsrp) and various cell cycle proteins, including cyclins E1 and L2, have been found in growth cones [8]. Conceivably, retrograde trafficking of these locally translated proteins may enable signalling at the growth cone to influence nuclear events.

## 9. Can axons synthesize membrane and secreted proteins?

Another intriguing finding of the transcriptome-wide analyses is the large number of transcripts encoding transmembrane or secreted proteins, which make up about 13 per cent of the mRNAs contained in growth cones [8]. Previous studies had already identified transcripts encoding transmembrane proteins in axons [44,45,76]. An analysis of the second-generation axonal libraries further revealed that axons contain transcripts encoding several classes of transmembrane proteins, including cell adhesion molecules (integrins, protocadherins) and guidance receptors (EphB4, Nrp2) [8]. This indicates that local translation might function to alter the cell adhesion properties of the axons and to confer responsiveness to extracellular signalling molecules such as guidance cues [7,8]. Moreover, transcripts encoding secreted proteins such as guidance molecules (semaphorins, ephrins), growth factors (such as BMP1, CTGF, FGF) and components and regulators of the extracellular matrix (collagen, TIMP3) are also found in axons [7,8], suggesting that local translation plays an active role in modulating the composition of the extracellular environment by influencing the make-up of proteins secreted from growth cones.

Although these studies suggest that transcripts encoding transmembrane proteins are a major component of the axonal transcriptome, it remains unclear whether these proteins can actually be translated and properly targeted within axons. Our general understanding of the organellar structures and the mechanisms for membrane targeting of newly translated proteins has been derived from studies in non-neuronal cells, which may not apply to axonal growth cones. The targeting of many transmembrane and secreted proteins involves the recognition of a signal peptide in the nascent protein being synthesized on the ribosome [77]. The ribosome–protein complex is then targeted to the endoplasmic reticulum (ER), where the protein is either secreted into the lumen or inserted into the membrane, in a process that requires specific ER proteins that control the topology of the nascent protein [77]. Once folded, the luminal domains of these proteins are targeted for specific modifications, such as disulphide bond formation and glycosylation, which are often essential for protein function. These modifications require both the ER and the Golgi apparatus, as well as a host of proteins within the lumen of these organelles [78]. However, none of the classical structures indicative of ER or Golgi needed for these diverse processes have been detected by electron microscopy in mouse myelinated axons [79].

Intriguingly, recent studies have identified ER- and Golgi-like structures in axons. Staining with ER- and Golgi-specific dyes revealed discontinuous and punctate labelling along the axon, raising the possibility that non-canonical versions of these organelles may exist in nerve terminals [80]. These structures contained markers of the secretory system such as SRP54, ribophorinII, TRAPalpha, KDEL, GM130 and TGN38 [80]. Similar structures were not found in mature hippocampal axons [81]. Although these structures appear to be small relative to the size of the ER/Golgi system in the cell body, studies have shown that small amounts of Golgi are sufficient for proper protein targeting to the membrane [82]. Moreover, these axonal structures resemble dendritic ‘Golgi outposts’ that have been identified in dendrites of rat hippocampal cells and have roles in dendritic secretory trafficking [81]. Although some markers have been identified in these axonal structures, a systematic analysis of the expression of the different proteins that are required for protein insertion in membranes and post-translational modifications has not been reported. Indeed, it is possible that these axonal Golgi-like structures may not actually function in the processing of locally synthesized proteins. For example, certain transmembrane proteins, such as the Trk receptors, are trafficked to axons in post-Golgi (exocytic) vesicles [83,84]. These vesicles may therefore contain Golgi markers but not be fully functional Golgi-like structures. Further biochemical studies are needed to determine whether axons contain functional ‘axonal Golgi outposts’.

## 10. Conclusions and future directions

**Figure 2.**
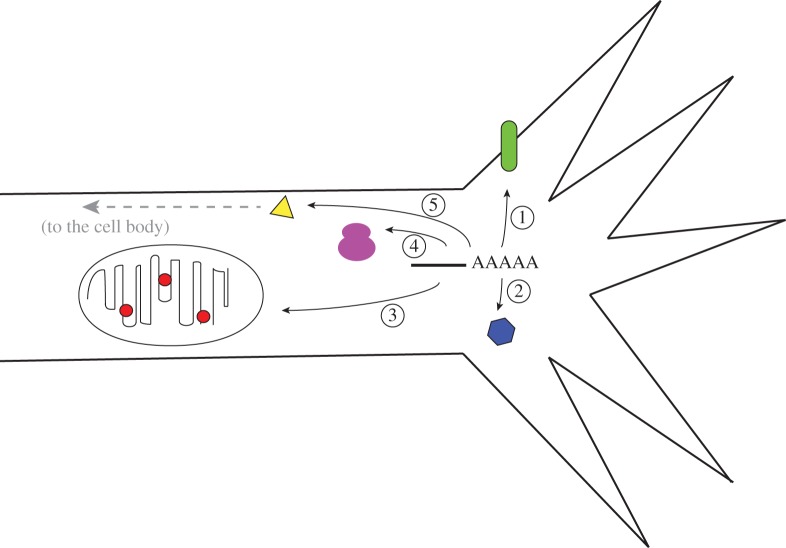
A diagram summarizing the intra-cellular pathways that may utilize local translation of axonal mRNAs. Axonal mRNAs may lead to the local production of transmembrane and extracellular proteins (1), proteins that function locally within axons and growth cones (2), that are targeted to mitochondria (3) or ribosomes (4) or that are retrogradely trafficked to the cell body (5).

The recent large-scale profiling studies of axons have highlighted the complexity of the axonal transcriptome and have identified novel mRNAs that are likely to have functional roles in various aspects of axonal biology (figure 2). These discoveries point to new or previously unappreciated roles for local translation and raise important new avenues for investigation. We highlight some of these in the upcoming sections.

### 10.1. What transcripts are found in the axons of living animals?

The studies that have sought to determine the roles of local translation have largely focused on cultured neurons. Therefore, it is possible that the phenomenon of mRNA trafficking to axons could reflect an adaptation to the culturing environment. Furthermore, the specific mRNAs that are detected by library approaches may be influenced by the culturing conditions. Recent studies have provided evidence for the physiological occurrence of axonal targeting of mRNA. Recently, the Twiss group described a genetically engineered mouse that expresses an RNA that interferes with the function of ZBP-1, a protein that is important for trafficking β-actin mRNA to axons [85,86]. These mice exhibit defective axonal regeneration, supporting the idea that local translation has roles in this process. In another study, our group showed that BDNF induces the intra-axonal synthesis of SMAD1/5/8 [33]. In embryos deficient in BDNF, SMAD1/5/8 is depleted from axons, but not from cell bodies, supporting the idea that BDNF physiologically regulates intra-axonal protein synthesis of SMAD1/5/8. However, despite these few examples, future studies are needed to ascertain whether the various roles ascribed to axonal mRNAs based on studies in cultured neurons can be validated *in vivo*.

To understand the role of local translation *in vivo*, it will be important to determine which mRNAs are localized to actively growing axons during embryogenesis, in adult animals, and after axonal injury. New genetically engineered animals may help to address this question. We recently described the use of the EGFP-L10a mouse [87] to explore ribosome localization in adult and regenerating axons in animals [88]. These mice express a fluorescent form of the ribosomal protein L10a under the control of the *Glt25d2* promoter that drives transgene expression in layer 5b cortical neurons [89]. Using these mice, we found EGFP-L10a labelling at the nodes of Ranvier in uninjured mice, and prominent labelling in the growth cones of regenerating spinal axons [88]. These transgenic mice are especially useful to selectively image ribosomes in long projection axons, without the problem of detecting ribosomes from nearby non-axonal tissue. This general approach can also be used to selectively harvest ribosomes and their bound mRNAs from axons. To do this, the corticospinal tract can be dissected and subjected to anti-EGFP immunoprecipitation. This will result in the selective recovery of axonal ribosomes since EGFP-L10a is expressed only in cortical neurons and their descending axons in the spinal cord. Ribosomes from the surrounding myelin or other supporting cells will not be recovered because they will not express EGFP-L10a. This approach can be used to identify transcripts bound to ribosomes in normal adult corticospinal tract axons, regenerating axons after injury or even in growing axons during development. The use of different promoters to drive the expression of the transgene could potentially allow the isolation of axonal ribosomes from other neuronal populations, as long as the axons are physically distant from the cell bodies that express EGFP-L10a. These experiments could reveal which mRNAs are localized to axons during pathfinding and in other physiological contexts.

The use of *in vivo* systems will also be important to better understand the role of the supporting glial cells in influencing intra-axonal translation. A recent study using a ribosomal protein reporter found that Schwann cells can transfer polyribosomes and, potentially, mRNAs to axons severed from cell bodies [90]. This finding suggests that neighbouring glial cells can directly modulate axonal gene expression by regulating the content of axons. It will be important to understand whether the exchange of mRNA and ribosomes between glia and neurons occurs only in response to axonal injury or whether it is a constitutive physiological pathway used to influence axonal biology.

### 10.2. Are transcripts that require endoplasmic reticulum and Golgi processing synthesized and targeted in axons?

A major challenge in understanding whether axons can produce secreted and membrane-bound proteins is to demonstrate that these proteins can be locally processed in the absence of typical ER and Golgi structures. Despite the apparent absence of ER and Golgi, several studies have suggested that axon growth cones may have ER/Golgi-like functionality. The strongest data supporting this idea were the demonstration of axonal protein glycosylation by Flanagan and co-workers [46]. These investigators examined the fate of placental alkaline phosphatase (PLAP) transcripts transduced into chick retinal axons using Sindbis virus, an RNA virus that is translated by the host ribosomes [91]. PLAP is a glycosyl phosphatidylinositol (GPI)-anchored protein that is enzymatically active only when exported to an extracellular location [46]. Addition of the GPI anchor, which is typically thought to occur in the ER, allows the docking of the protein to the outer surface of the cell membrane. Therefore, detection of surface immunoreactivity for the PLAP reporter could serve as an indicator of GPI anchor addition. Indeed, heterologous PLAP expression resulted in detectable surface phosphatase activity, suggesting that PLAP can be locally glycosylated and inserted in the membrane [46]. Interestingly, a review of the second-generation mRNA libraries shows that mRNA for at least one well-characterized GPI-anchored protein, NCAM, can be found in axons.

However, it is difficult to extrapolate from the PLAP experiment whether the more complex patterns of membrane insertion or glycosylation seen in most other receptor proteins can occur in axons. Experiments monitoring the trafficking of locally translated membrane-targeted proteins during different stages of protein processing in axons are needed. It will be important to directly measure axonal glycosylation and determine whether it results in similar patterns of glycosylation complexity as those seen in cell bodies. To further establish the extent to which axonally synthesized proteins are locally glycosylated, recently described azide- and alkyne-tagged glycosylation precursors labelled with ‘click chemistry’ [92] might allow the monitoring of glycosylation in isolated axons or in compartmentalized but intact neurons. These experiments will provide evidence for the presence of functional ER- and Golgi-like structures in axons.

### 10.3. Do axonal transcripts have different sequence features than transcripts in cell bodies?

Although many of the transcriptomic studies have identified novel mRNAs that are present in axons, the specific transcript variants that are localized are not yet known. All transcript are susceptible to alternative splicing and it is estimated that more than 50 per cent mammalian transcripts use alternative poly(A) sites [93], which influences the 3′UTR length. In addition, in some cases, the use of alternative promoter can lead to different 5′UTRs. Techniques such as next-generation sequencing (NGS) are particularly useful for determining transcript structures [94], and therefore may identify specific variants that are selectively trafficked to axons. Indeed, recent studies have provided specific examples of distinct variants of the same transcript that are differentially transported to axons [22,23,28]. Application of NGS to the axonal transcriptome will be useful to efficiently identify transcript variants that are preferentially enriched in axons. Ultimately, this may help in the identification of sequences or elements that confer axonal targeting.

These variants may also be differentially regulated, as variant-specific sequences may contain microRNA-binding sites or sites for the association of RNA-binding proteins. The potential role for microRNAs in regulating local translation was suggested by the finding that proteins involved in microRNA-mediated translational silencing are present in embryonic axons, and that introduction of small interfering RNA into axons leads to degradation of target mRNAs [40]. Several microRNAs have since been shown to be enriched in distal axons [95], and miR-9 has been shown to locally regulate BDNF-dependent axon extension and branching [96].

### 10.4. How do the diverse axonal transcripts get targeted to axons?

Only a few *cis*-acting elements have been identified that target mRNAs to axons. Of these, the β-actin zipcode sequence is one of the best characterized [97,98]. This element, a conserved bipartite sequence present at the beginning of the β-actin 3′UTR [97,99], has been shown to interact with the RNA-binding protein ZBP-1 to mediate axonal localization of β-actin [4,100]. However, the canonical zipcode sequence is found only in a limited number of transcripts [99], raising the question of what mechanisms are used to target other mRNAs to axons. One possibility is that the binding of an mRNA to ZBP-1 is not exclusively determined by the presence of the canonical zipcode sequence in the mRNA. Rather, specific recognition of the mRNA might depend on the secondary and tertiary structure that the mRNA assumes when folded. It is therefore possible that ZBP-1 has many more targets that can be predicted using current algorithms. Indeed, a recent report found that competing away ZBP-1 by overexpressing a β-actin 3′UTR transgene reduces not only axonal levels of β-actin, but also *GAP43* mRNA, which lacks a canonical zipcode [85]. Further studies are needed to understand which *cis-*acting elements and proteins determine the axonal localization of mRNAs that do not use ZBP-1.

The axonal mRNA profiling studies can also offer insights into some of the potential mechanisms regulating axonal mRNA transport. For example, these studies found that mRNAs for ribosomal proteins are enriched in axons. A common feature of all ribosomal transcripts is the *cis*-element at the beginning of their 5′UTR known as 5′-terminal oligopyrimidine tract (5′-TOP), which consists of a C followed by a tract of 6–10 pyrimidines [101]. It is possible that this shared feature accounts for the selective enrichment of the ribosomal transcripts in axons. Consistent with this idea, other transcripts that contain 5′-TOP sequences, such as all the five elongation factors and the initiation factors eIF3e, eIF3f and eIF3h [102] are also enriched in axons. How could 5′-TOP sequences be targeted to axons? The La protein, which has previously been shown to bind 5′-TOP sequences [103], is known to be trafficked into axons [104], making La a candidate for axonal transport of these mRNAs.

In addition to ribosomal transcripts, it is also unknown how transcripts encoding mitochondrial proteins are targeted to axons. Transcripts encoding mitochondrial proteins have not been shown to share a common sequence motif, which might be implicated in axonal targeting. However, these transcripts associate with the outer surface of the mitochondria [105,106]. How could this explain the axonal localization of mitochondrial mRNAs? One model is that these mRNAs are transported to axons together with the mitochondria. mRNA association with the mitochondrial surface is co-translational, and mediated by a mitochondrial targeting signal on the nascent protein that is recognized by receptors on the mitochondrial outer surface [107]. As with proteins targeted to the ER, the complex of the nascent mitochondrial protein, its corresponding mRNA and ribosomes are bound in close proximity to mitochondrial pores that mediate the translocation of the newly translated protein inside the mitochondria [107].

### 10.5. Why are only some axonal proteins synthesized locally?

One of the major insights revealed from the axonal transcriptome libraries is that many proteins that are known to be present in axons are not synthesized locally. For example, the components of the PAR polarity complex have all been localized to distal axons and growth cones by immunofluorescence [16]. However, axonal mRNA for PAR-6 and aPKC*ζ* was not detected by FISH [16] and did not appear in any of the axonal mRNA libraries. Thus, these proteins were most likely synthesized in the cell body and transported to the growth cone. In contrast, PAR3, another component of the PAR complex, is locally synthesized from axonally localized *PAR3* transcripts [16]. The idea that only a subset of axonal proteins are synthesized locally is supported by metabolic labelling experiments using axonally applied [^35^S]-methionine/cysteine [108]. These studies showed that only approximately 5–10% of neuronal proteins are locally synthesized in axons, while the majority of axonal proteins are synthesized in cell bodies and anterogradely transported into axons. Thus, only certain proteins in axons are locally translated while other proteins are derived from the cell body. These findings raise the question of the underlying logic behind why some proteins are locally synthesized and why others are made in the cell body and subsequently transported to axons. Conceivably, certain common regulatory or functional aspects of locally translated proteins may explain why these proteins are preferentially translated within axons.

An additional consideration is that some axonal proteins may derive from both local synthesis and transport from the cell body. For example, selective application of protein synthesis inhibitors to axons showed that less than 1 per cent of the axonal actin and tubulin derive from local synthesis [109]. Why would both cell-body-derived and locally synthesized proteins both be needed in axons? One possibility is that the locally translated protein may have a different set of post-translational modifications than the cell-body-derived proteins, as has been suggested for the local synthesis of β-actin [9].

### 10.6. What is the significance of the low-abundance and ‘non-enriched’ axonal transcripts?

Most of the information on the roles and importance of local translation comes from studies that look at transcripts that are highly abundant in distal axons. However, the second-generation axonal libraries have also uncovered the presence of a subset of transcripts that localize to axons but have low abundance compared with other axonal mRNAs. It is possible that low-abundance transcripts represent mRNAs that are not actively targeted to axons but localize to axons through diffusion or other passive mechanisms, thus representing transcriptome ‘noise’. However, it is also possible that low-abundance or non-enriched transcripts are locally translated and are required for specific axonal functions. Indeed, β-actin transcripts are not enriched in axons [22], although local translation of β-actin has important roles in axonal turning [12,13]. Axon-specific knockdown will be a valuable tool to assess the roles of these non-enriched axonal transcripts.
